# Reward, salience, and attentional networks are activated by religious experience in devout Mormons

**DOI:** 10.1080/17470919.2016.1257437

**Published:** 2016-11-29

**Authors:** Michael A. Ferguson, Jared A. Nielsen, Jace B. King, Li Dai, Danielle M. Giangrasso, Rachel Holman, Julie R. Korenberg, Jeffrey S. Anderson

**Affiliations:** a Department of Bioengineering, University of Utah, Salt Lake City, UT, USA; b Department of Psychiatry, Massachusetts General Hospital, Boston, MA, USA; c Department of Psychology and Center for Brain Science, Harvard University, Cambridge, MA, USA; d Interdepartmental Program in Neuroscience, University of Utah, Salt Lake City, UT, USA; e Department of Pediatrics, University of Utah, Salt Lake City, UT, USA; f Department of Radiology, University of Utah, Salt Lake City, UT, USA

**Keywords:** Religious neuroscience, reward, spiritual experiences, functional MRI

## Abstract

High-level cognitive and emotional experience arises from brain activity, but the specific brain substrates for religious and spiritual euphoria remain unclear. We demonstrate using functional magnetic resonance imaging scans in 19 devout Mormons that a recognizable feeling central to their devotional practice was reproducibly associated with activation in nucleus accumbens, ventromedial prefrontal cortex, and frontal attentional regions. Nucleus accumbens activation preceded peak spiritual feelings by 1–3 s and was replicated in four separate tasks. Attentional activation in the anterior cingulate and frontal eye fields was greater in the right hemisphere. The association of abstract ideas and brain reward circuitry may interact with frontal attentional and emotive salience processing, suggesting a mechanism whereby doctrinal concepts may come to be intrinsically rewarding and motivate behavior in religious individuals.

## Introduction

Religious and spiritual experiences share common phenomenological elements across cultures and theistic faith traditions, including a profound sense of elevated mood (euphoria), noetic insight, ineffability, and a sense of integration within oneself and with others (James, 1902). Similar feelings are described in association with romantic and parental love, reward, and drug-induced euphoric states, suggesting common neural mechanisms for these experiences. Among such rewarding stimuli, religious experience uniquely contributes to establishment of social systems with far-reaching consequences for pro- and antisocial behaviors (Decety et al., 2015; Shariff & Norenzayan, 2007). Despite the reported impact of religious experience in the lives of more than 5.8 billion religiously affiliated individuals worldwide (Hackett & Grim, 2012), even basic questions about brain networks engaged by religious experience remain unclear.

Hypotheses about the neurobiology underlying religious experience are discordant in the literature. A thread of research arising from the pathophysiology of hyperreligiosity in epilepsy and schizophrenia suggested that religious ideation and experience arises from temporal lobe structures (Devinsky & Lai, 2008; Dewhurst & Beard, 1970; Trimble & Freeman, 2006). Other reports have synthesized studies from the literature to suggest a right lateralized network involving amygdala, temporal, and frontal cortex as underlying religious experience (McNamara, 2009b). Yet, others have hypothesized involvement of the ventral striatum and nucleus accumbens (Deeley, 2004; McNamara, 2009a; Schjoedt, Stodkilde-Jorgensen, Geertz, & Roepstorff, 2009).

Although to date no study has shown activation in the ventral striatum, the involvement of these regions in religious experience may contribute to the rewarding aspects of religious belief and adherence to religious doctrine and leaders. Activation of the caudate head during prayer (Schjodt, Stodkilde-Jorgensen, Geertz, & Roepstorff, 2008) and during recollection of a mystical experience (Beauregard & Paquette, 2006) has been observed; however, the caudate contributes to many neurobiological processes including language, cognition, perception, and motor function, due to its involvement in multiple domain-specific corticostriatal circuits (Grahn, Parkinson, & Owen, 2008; Robinson et al., 2012).

Although this work has begun to identify putative brain regions involved in religious experience, descriptions of religious experience are broad, encompassing elements of emotion, salience, language, attention, arousal, memory, and social and moral cognition that are difficult to isolate within traditional categories of brain function. Therefore, a more comprehensive account of whole brain activity during religious experience is needed. Additionally, it is unknown whether subjective feelings attributed to religious or spiritual experience are represented by neural activity in similar brain networks across individuals, or whether brain activation may be heterogeneous or idiosyncratic from individual to individual.

Previous reports have examined neural correlates of religious experience in Franciscan nuns (A. Newberg, Pourdehnad, Alavi, & d’Aquili, 2003), Pentecostal women with glossolalia (A. B. Newberg, Wintering, Morgan, & Waldman, 2006), Carmelite nuns (Beauregard & Paquette, 2006), German-Christian evangelicals (Azari et al., 2001), Danish-Christians (Schjodt et al., 2008; Schjoedt, Stodkilde-Jorgensen, Geertz, Lund, & Roepstorff, 2011; Schjoedt et al., 2009), Brazilian mediums (Peres, Moreira-Almeida, Caixeta, Leao, & Newberg, 2012), and Chinese-Christians (Han et al., 2008). These studies suggest the need for larger samples, reproducible ecologically valid stimuli, and study designs that can assess both sustained and temporally fluctuating euphoric states. A larger literature has examined the neural correlates of contemplative and mindfulness practices (Brefczynski-Lewis, Lutz, Schaefer, Levinson, & Davidson, 2007; Lutz, Greischar, Rawlings, Ricard, & Davidson, 2004; Tang, Holzel, & Posner, 2015), with hypothesized neural correlates of modulation of attention, regulation of emotion, and alterations in self-awareness (Tang et al., 2015). Distributed cortical networks have been suggested to contribute to representations of belief in religious contexts (Harris et al., 2009; Harris, Sheth, & Cohen, 2008).

Evoked feelings during religious practice have pronounced effects on social behavior, health, and religious commitment. Feelings of peace and joy during prayer have been associated with increased future religious commitment and improved sleep quality, while other religious experiences such as glossolalia, perception of having prayers answered, and perception of miraculous healing showed no similar effect in a longitudinal analysis (Hui et al., 2015). Frequent spiritual or religious experience has also been associated with enhanced quality of life and positive psychosocial outcomes (Underwood & Teresi, 2002). In contrast, religiously motivated violence is frequently encountered in society (Rowley, 2014), and there is poor understanding of what motivates attachment to religious ideas and leaders who promote violent extremism among followers.

A neuroscience of religious and spiritual experience is a key step for understanding the motivation of religious behavior and health effects of religious practice across communities. We selected a Mormon population for studying subjective religious euphoria because of the centrality of charismatic religious joy (colloquially, “feeling the Spirit”) in both Mormon theology and practice, and the high frequency with which adherents to the faith report experiencing these phenomena in their daily lives. Identifying spiritual and religious experience in oneself and teaching this ability to others is a primary focus of conversion and missionary efforts in the Mormon Church, and a core daily activity during mission service is devotional practice of scripture study and prayer during which an individual learns to recognize such spiritual feelings. The goal of this study was to identify whether self-identified spiritual feelings in these individuals were reproducibly associated with the activation of specific neural circuits. Our hypothesis was that reward circuits of the ventral striatum would be activated during self-identified spiritual feelings, suggesting a mechanism whereby reward-driven religious experience could contribute toward the establishment and maintenance of religious belief and attachment to religious leaders.

## Materials and methods

### Participant characteristics

Nineteen young adult participants aged 27.4 ± 3.6 years, 7 female, 12 male, were among 44 potential participants recruited through local media coverage of the study announcement. All participants gave informed consent to participate under guidelines approved by the University of Utah Institutional Review Board. Participants were selected from among the 44 volunteers on the basis of younger age, female sex to achieve a more gender-balanced sample (of 44 participants, 15 were female), and frequency of worship service attendance and private devotional practice. Selection criteria were designed to recruit a subject sample more likely to experience recognizable spiritual feelings in a controlled environment. Specifically, selection criteria excluded subjects for imaging that were over 40 years old, that did not report average weekly church attendance, and that did not report on self-assessment questionnaire that subjects experienced spiritual feelings in their daily life. Demographics of both groups of subjects are reported in Table 1. All 44 participants completed a period of 1.5–2 years of missionary service for the Mormon Church as a young adult.

Twenty-two subjects were selected for neuroimaging. Of these 22 subjects, 2 pilot subjects were scanned using an abbreviated protocol that included only resting state and prayer functional magnetic resonance imaging (fMRI) sessions and a third subject was not analyzed in the study due to a technical malfunction of the MRI scanner halfway through the scan that required rebooting the scanner and aborting the scan. Demographic characteristics of the 19 subjects for whom functional imaging is reported in this study as well as for remaining subject sample that was not imaged are shown in Table 1.

### Experimental paradigm

The study was designed to measure the neurobiological substrates of religious experience in devout Mormons, and several stimulation paradigms were employed to elicit such spiritual and religious feeling, using ecological stimuli customized to Mormon religious experience. Within a Mormon religious tradition, spiritual and religious practice consists of prayer, scripture study, audio-visual presentations of religiously themed music and teachings of church leaders, and study of teachings of religious authorities, and all of these elements were incorporated into a session of about 1 h, a typical length for a Mormon religious service. Participants were scanned using multiband fMRI imaging during religiously evocative stimuli consisting of prayer, scripture reading, quotations from Latter-day Saints (LDS) and non-LDS religious authorities, and audiovisual stimuli. Stimuli were chosen to replicate typical patterns of religious practice common among adherents.

The experimental design treats each participant as their own control by comparing points in time where they have relatively stronger or weaker religious experience. To address the critical issue of replicability of the results, independent paradigms were used that allowed for replication of the results with similar and differing stimuli, and affording both temporal and spatial characterization of religious experience. Each exam consisted of a 1-h imaging paradigm that included structural MP2RAGE imaging followed by the following seven functional paradigms:

Resting state (6 min): Participants were told to close their eyes but remain awake, letting thoughts pass through their mind without focusing on any particular mental activity.

Audiovisual control (6 min): Participants watched audiovisual stimuli consisting of the statistical report from a recent general conference of the Mormon Church detailing membership and financial reports from a recent internal audit.

Quotations (8 min): Participants were shown 24 quotations that they were instructed were from “LDS and world religious authorities.” A brief, Christian-themed quotation was shown in black on a gray screen, underneath which attribution was given to one of three Mormon authorities (Thomas Monson, Dieter Uchtdorf, and Jeffrey Holland) or one of three non-Mormon authorities (Pope Francis, Billy Graham, and Desmond Tutu). A small photograph of the authority was shown to the right of their name. All quotations were selected from the writings of C.S. Lewis and randomly attributed with counterbalancing to one of the six authorities. Participants were not informed of the incorrect attributions. An additional 24 quotations (also from C.S. Lewis) were shown in a repeat quotations task at the end of the scan, and the 24 quotations shown in the first task were randomly selected from the 48 quotes for each subject. Including quotations both from in-group and out-group religious authorities helps to address concerns of social desirability, whereby subjects may be more likely to report spiritual feelings in response to statements by their own religious leaders.

Each quotation was displayed for 10 s, followed by a question “Are you feeling the Spirit?” and responses “1 – not feeling; 2 – moderately feeling; 3 – strongly feeling; and 4 – very strongly feeling,” displayed for 5 s during which participants were instructed prior to the scan to press a button corresponding to their selection. This was then followed by an additional question “How spiritually meaningful is this quotation to you?” with responses “1 – less spiritually meaningful; 2 – moderately spiritually meaningful; 3 – very spiritually meaningful; and 4 – deeply spiritually meaningful,” also displayed for 5 s. Stimuli were presented with E-Prime software and synchronized to the beginning of the BOLD acquisition after 10 s of discarded images.

Prayer (6 min): Participants were instructed to close their eyes and pray according to their typical devotional practice.

Scripture reading (8 min): Participants were shown short passages from the Book of Mormon, the principal religious text for Mormons, in black text on a gray screen, selected from among “Scripture Mastery” lists produced by the Mormon Church Educational System that Mormon missionaries read and sometimes memorize during missionary service. Without exception in post-scan debriefing, participants indicated that they were familiar with all of the scriptural passages shown.

Each scriptural passage was displayed for 20 s, followed by a question “Are you feeling the Spirit?” and responses “1 – not feeling; 2 – moderately feeling; 3 – strongly feeling; and 4 – very strongly feeling,” displayed for 5 s during which participants were instructed prior to the scan to press a button corresponding to their selection. This was then followed by an additional question “How spiritually meaningful is this passage to you?” with responses “1 – less spiritually meaningful; 2 – moderately spiritually meaningful; 3 – very spiritually meaningful; and 4 – deeply spiritually meaningful,” also displayed for 5 s. Stimuli were presented with E-Prime software and synchronized to the beginning of the BOLD acquisition after 10 s of discarded images.

Audiovisual stimuli (12 min): Two separate blocks of 6 min were obtained during presentation of two “Mormon Messages” videos created by the LDS Church (https://www.mormonchannel.org/watch/series/mormon-messages) that contain religiously evocative content. Videos consisted of short excerpts from speeches by Mormon Church authorities, imagery of artistic renderings of Biblical scenes, testimonials of church members from multiple cultures, and pictures of children and family scenes. Stimuli were presented using E-Prime software, with the start of the video clips synchronized to the beginning of the BOLD acquisition after 10 s of discarded images.

Participants were instructed to press a button (one button only) when they were feeling peak spiritual feelings, and that they could press the button more frequently when the feeling was more intense.

Quotations (8 min): The remaining 24 quotations were displayed using the same design described above. By using similar but distinct stimuli to the first quotations paradigm, we were able to use the two tasks as a replication experiment as well as to determine if differences in religious experience were seen early and late during a sustained period of religious stimulation.

### Behavioral measures

In addition to neuroimaging data, behavioral self-report data from questionnaires were completed by each subject prior to the imaging session. Two metrics of interest were analyzed in conjunction with the imaging data. Dimensions of religiosity are a normed metric of religiosity specific to Mormon belief and practice with subscales of religious behavior, commitment, and belief, and two modes of religiosity: personal and institutional (Cornwall, Albrecht, Cunningham, & Pitcher, 1986). The moral foundations questionnaire is a normed metric that evaluates relative moral values in five domains: harm/care, fairness/reciprocity, in-group/loyalty, authority/respect, and purity/sanctity (Graham et al., 2011). Personality traits were assessed using the NEO Five Factor Inventory (McCrae & Costa, 2010). Summary results from the participant sample are displayed in Table 1. Subjects selected for imaging and those not selected for imaging showed no significant differences in religiosity or for scores on the moral foundations questionnaire, but the two groups differed with the imaging group showing higher agreeableness (*p* = .010, two-tailed *t*-test) and a trend toward decreased neuroticism (*p* = .07) on the five factor personality model when compared with subjects not included in the imaging sample.

**Figure 1:**
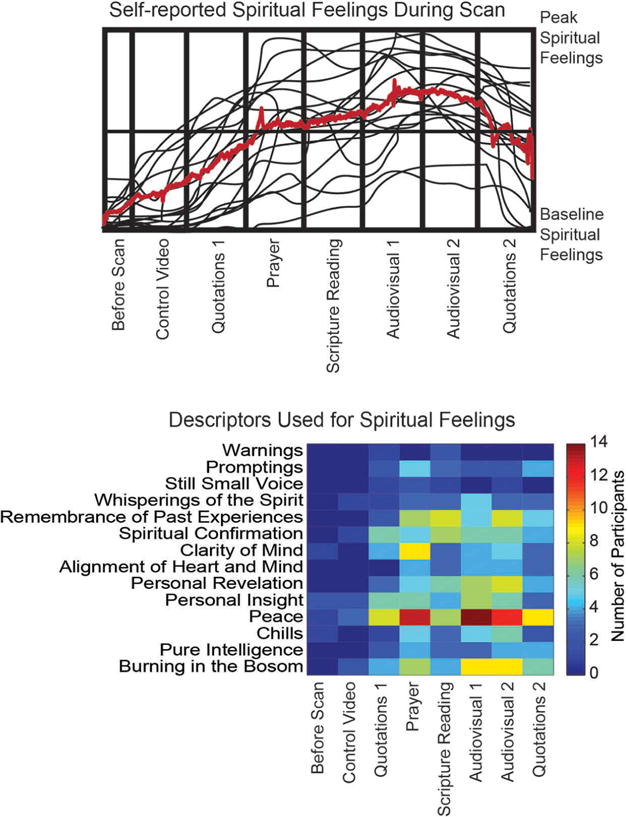
Subjective spiritual feelings throughout the imaging session. Above: Digitized hand-drawn traces for each subject of relative spiritual feelings during the MRI scan session compared to baseline feelings and peak spiritual feelings experienced during private devotional practice and worship services. Traces were drawn during a debriefing following the MRI scan. Below: Following the scan session, participants selected from among 14 terms commonly used in addresses from Mormon leaders which terms best described spiritual feelings they felt during each section of the MRI scan session.

A debriefing was performed following each subject’s imaging session that included a qualitative question and answer session about the experience of the subject as well as a more formalized characterization of the types of religious experience felt during each part of the scan. Subjects were given a blank grid including on the *x*-axis each of the components of the scan and the *y*-axis a range from “Baseline Spiritual Feelings” to “Peak Spiritual Feelings” and instructed to compare the overall level of spiritual experience during the scan to what they experience during worship services, temple attendance, and private devotional practice. They were also asked to select for each portion of the scan what phrases might best describe the religious feelings they experienced, selected from among 15 phrases commonly used in addresses over the last 10 years by Church leaders to describe “feeling the Spirit” in addresses at the Church General Conference. These results are shown in Figure 1 and suggest that the types of religious experience felt in the scan session conformed in quality and magnitude with meaningful experiences in private and group religious practice.

### Autonomic physiological waveforms

During all functional imaging acquisitions, heart rate and respiratory rate waveforms were recorded, synchronized to the onset of BOLD imaging. The median heart rate and number of respirations were extracted for each individual during the 15 s following the onset of the display of each quote and during 25 s following the onset of each scriptural passage. These values were then correlated with the subjects’ subjective rating of the extent to which they were “feeling the Spirit” during each stimulus block.

### Image acquisition

Images were obtained on a Siemens Trio 3 T MRI scanner. A 32 channel head coil (Siemens) was used for acquisition. Structural images consisted of MP2RAGE sequence (Siemens) that included T1, T2, and MP2RAGE images. Sequence parameters were repetition time: 5000 ms, echo time: 2.91 ms, spatial resolution: 1 × 1 × 1 mm.

Functional images were acquired using multiband BOLD sequence acquired from the University of Minnesota (Setsompop et al., 2012) and configured with the following sequence parameters: repetition time: 730 ms, echo time: 30 ms, spatial resolution: 2 × 2 × 2 mm, multiband factor: 8.

### Image processing

**Figure 2:**
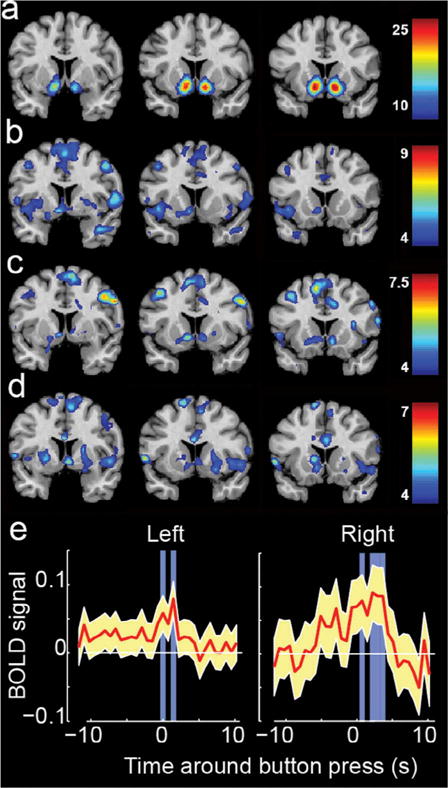
Brain activation associated with “feeling the Spirit” across multiple task paradigms. (a) Regions associated with the term “reward” in the functional neuroimaging literature. (b–d) Brain activation associated with “feeling the Spirit” while viewing quotations (b,d) or scriptural passages (c). Color scale shows *t*-statistic, with significant regions satisfying *q* < .05, False-discovery rate corrected. (e) Left and right nucleus accumbens activity before and after moments of strong spiritual feeling during audiovisual stimuli. Blue regions show *p* < 0.05 for activity greater than the mean.

Structural images were processed using the FreeSurfer imaging analysis environment (v5.3.0), which is documented and freely available for download (http://surfer.nmr.mgh.harvard.edu/). Detailed description of the morphometric procedures used in the FreeSurfer pipeline can be found on the FreeSurfer website. Images were first corrected for any head motion, followed by removal of non-brain tissue removal using a hybrid watershed/surface deformation procedure, automated transformation to Talairach space, deep gray matter and subcortical white matter segmentations intensity normalization, tessellation of the white and gray matter boundary, automated topology correction, and surface deformation following intensity gradients which leads to border placement of gray/cerebrospinal fluid and gray/white at the location with the greatest intensity shift, thereby defining tissue class transition (Dale, Fischl, & Sereno, 1999; Fischl et al., 2002; Fischl, Sereno, & Dale, 1999). Automated segmentation was manually inspected for goodness of fit with manual adjustment of the gray/white boundary as needed. FreeSurfer images in MNI space containing segmentation of the nucleus accumbens were used to define left NA and right NA regions of interest used in the analysis of the audiovisual stimuli (Figure 2(e)).

Functional images were preprocessed using SPM12b software (Wellcome Department of Imaging Neuroscience, London, UK) in MATLAB (MathWorks, Natick, MA). The following image processing pipeline was performed for all BOLD sequences retaining 2 × 2 × 2 mm spatial resolution:

- Realign (Estimate and Reslice)
- Coregister to T1 image from MP2RAGE sequence
- Normalize T1 image to MNI template with parameters applied to BOLD images
- Spatial smoothing (6-mm FWHM kernel)

First-level statistical analysis included selecting as contrasts epochs displaying quotation or scriptural stimuli, with covariates including the “feeling the Spirit” rating entered by button press following the quote for each block and the “Meaningful” rating entered by button press following the block. Motion parameters from coregistration step were included as regressors in the model.

**Figure 3:**
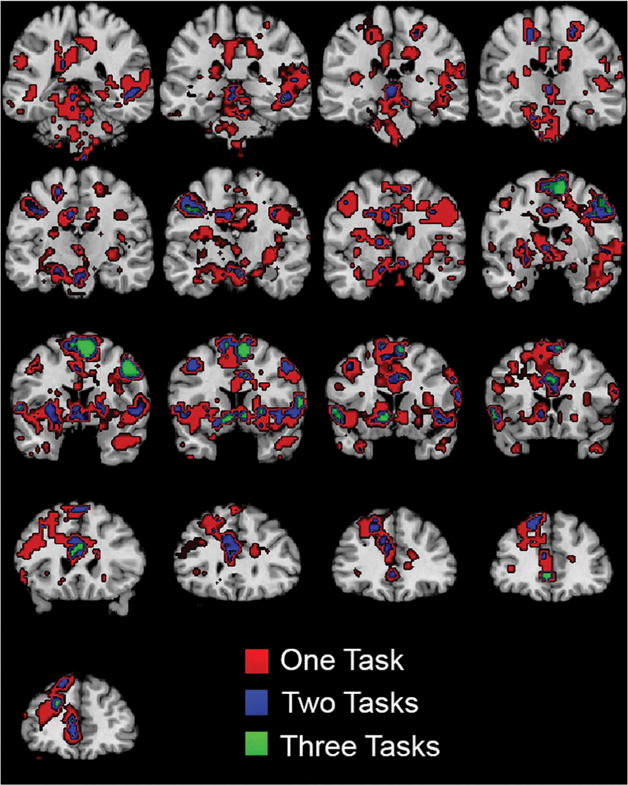
Conjunction analysis shows activation associated with “feeling the Spirit” during all three tasks. Colors show significant activation during 1, 2, or 3 tasks, each thresholded at a *t*-statistic of 3.69, corresponding to a *p*-value < .001. Coronal images are shown with subject left on image left, with MNI slices ranging at 5-mm intervals from *y* = −40 to *y* = 40.

Second-level random effects statistical analysis included the activation map image for each subject from first-level analysis for “feeling the Spirit” contrast, with age and sex of participants as covariates. A one-sample *t*-test was used as a statistical outcome measure, with cluster-defining threshold of *p* < .001 and false-discovery rate cluster-corrected results considered significant in analysis for each of the three tasks (Quotations Initial, Scripture Reading, Quotations Final) analyzed separately. Results for this step are displayed in Figure 2(b–d). Significant clusters for each of the three tasks are listed in Table 2, and coronal images showing slices at 5 mm increments throughout the brain are shown in Figure 3, displayed at a threshold of *p* < .001 for conjunction analysis.

For analysis of audiovisual stimuli, the first 10 s and last 10 s of each 6-min acquisition were discarded, time series were detrended, and images were analyzed using a general linear model using event-related design with 5-s epochs prior to each button press as events and motion parameters as regressors. Second-level analysis was performed across subjects as above with age and sex as covariates.

Subsequently, BOLD time series from left and right nucleus accumbens (as defined by automated FreeSurfer segmentation in each subject) were extracted for a time interval that included 10 s prior to and following each button press. Prior to extraction, the entire time series of each audiovisual acquisition was normalized by a linear detrend of the time series and subtracting the mean and dividing by the standard deviation of the time series. The 20-s epochs surrounding each button press across all subjects were pooled and a two-tailed, one-sample *t*-test was performed for each time point corresponding to the repetition time of the scan (730 ms) in the 20-s epoch. Results of this step are shown in Figure 2(e).

## Results

We report data acquired from 19 young adult (age 27.4 ± 3.6 years, 7 female, details in Table 1) participants who have completed 1.5–2 years of voluntary full-time missionary service for the Church of Jesus Christ of LDS (also known as Mormon Church). Participants reported progressive, sustained subjective experience during the scan that was typical of feelings experienced during private devotional practice (Figure 1), superimposed with temporally fluctuating moments of more intense spiritual feeling. Self-report of subjective religious experience identified feelings of peace and physical sensations of warmth.

In three separate fMRI task acquisitions, each 8-min long, participants viewed quotations attributed to LDS and non-LDS religious authorities (two of the three acquisitions) or read passages from the Book of Mormon (remaining acquisition). After each 20 s stimulus, participants were asked how strongly they were “feeling the Spirit” (5 s response) and then how spiritually meaningful each quotation or scriptural passage was to them (5 s response), using a Likert scale from 1 (not feeling the Spirit or stimulus was not meaningful) to 4 (peak spiritual feelings or deeply meaningful stimulus). After image preprocessing, a block design general linear model was applied to epochs where stimuli were presented, using realignment parameters as covariates and ratings of “feeling the Spirit” and “meaningful” as contrasts. This approach allows separation of the cognitive effects of decision-making and button pressing, which occur after the stimulus presentation block, from the imaging contrast, which occurs during the presentation of and reflection on the stimuli, which occur during the stimulus presentation block.

For both quotations tasks (before and after religious priming) as well as for the scriptural passages task, activation during the presentation of quotes or scriptural passages showed reproducible correlations with subsequent ratings of how much participants were “feeling the Spirit.” Results are shown in Figure 2(b–d). In all three tasks, significant (false-discovery rate cluster corrected) activation was observed for the “feeling the Spirit,” but not “meaningful” contrasts across subjects in one or both nucleus accumbens as well as in several cortical regions. Across the three task acquisitions, significant activity was observed in three loci in the anterior cingulate cortex, supplementary motor area, and right frontal eye field for all three tasks (Table 2). Conjunction analysis in Figure 3 shows brain regions where greater spiritual feeling was associated with activation in one or more tasks in the group. To compare the activation loci in the ventral striatum with locations significantly associated with the term “reward” in the functional neuroimaging literature, a reverse inference is shown in Figure 2(a) from the NeuroSynth database (Yarkoni, Poldrack, Nichols, Van Essen, & Wager, 2011), indicating regions significantly likely to be activated in studies with the term “reward.”

Autonomic responses measured during tasks consisted of respiratory and plethysmographic waveforms. Heart rate and respiratory rate were calculated for each block in each of the quotations and scriptural passage tasks. Across the three scripture and quotes tasks, a positive correlation was found between participants’ subjective rating of how strongly they were “feeling the Spirit” and median heart rate (*r* = .119, *p* = .002) while a negative correlation was found with median respiratory rate (*r* = −.078, *p* = .048).

To evaluate the temporal precision of nucleus accumbens activation, structural MRI images were segmented using FreeSurfer software (version 5.3) (Fischl et al., 2002) to identify subject-specific locations of the nucleus accumbens. Activity from left and right nucleus accumbens in each subject was extracted from two 6-min audiovisual stimuli. During these stimuli, subjects were instructed to press a button when experiencing peak spiritual feelings, and to press more frequently if the feelings were more sustained or intense. Time series for 10 s of nucleus accumbens BOLD signal before and after each button press is displayed with average activity shown across button presses for all subjects in Figure 2(e). Significantly higher than average activity was observed in bilateral nucleus accumbens between 2 and 4 s following button presses. Given an expected hemodynamic response that peaks between 5 and 6 s, this likely corresponded to peak nucleus accumbens activity between 1 and 3 s prior to button presses.

## Discussion

We demonstrated in a group of devout Mormons that religious experience, identified as “feeling the Spirit,” was associated with consistent brain activation across individuals within bilateral nucleus accumbens, frontal attentional, and ventromedial prefrontal cortical loci. Brain regions associated with representation of reward were reproducibly activated in four distinct acquisitions using three experimental paradigms, with activation immediately preceding peak spiritual feelings identified by the participants by 1–3 s.

Activation of the striatum was consistent with findings of Schjodt et al. (2008) who observed dorsal striatal (right caudate head) activity in Danish-Christians in response to prayer stimuli. Bilateral caudate head activation was also seen in a study of Carmelite nuns recollecting a mystical experience (Beauregard & Paquette, 2006). Activation of the ventral striatum has not previously been reported in association with religious stimuli but is a hallmark of neurophysiological processes associated with reward and reinforcement (Balleine, Delgado, & Hikosaka, 2007; Tindell, Smith, Berridge, & Aldridge, 2009) with hypothesized links to religiosity (McNamara, 2009a; Schjoedt et al., 2009). Nucleus accumbens activity has been observed during several conditions of acutely positive affect including maternal and romantic love (Bartels & Zeki, 2000, 2004; Takahashi et al., 2015), appreciation of music (Blood & Zatorre, 2001; Salimpoor, Benovoy, Larcher, Dagher, & Zatorre, 2011; Salimpoor et al., 2013), and as a common pathway for chemically altered euphoric states associated with many drugs of abuse, including cocaine and methamphetamines (Pontieri, Tanda, & Di Chiara, 1995). Oxytocin physiology has also been linked to social processing of reward in the nucleus accumbens (Loth et al., 2014).

Genetic and behavioral factors influencing religiously motivated behavior appear related to dopamine metabolism and signaling. Inclination toward religious behavior and motivation has been associated with a polymorphism on the dopamine receptor gene DRD4 (Comings, Gonzales, Saucier, Johnson, & MacMurray, 2000; Sasaki et al., 2013). Acquired disorders of dopamine physiology also show links to religious behaviors. A study in patients with Parkinson disease has shown decreased frequency of private religious practices such as prayer and meditation in conjunction with disease progression (McNamara, Durso, & Brown, 2006). Dopamine physiology has been thought to underlie a heightened sense of importance and meaning surrounding otherwise insignificant stimuli in the context of psychosis, with accounts of patient experiences that overlap with characteristics of religious experience such as “coming alive,” “sharpening of the senses,” and attributing stimuli with “overwhelming significance”(Kapur, 2003). Nucleus accumbens activity has also been described in association with oxytocin (Olazabal & Young, 2006), opioid (Pecina & Berridge, 2005), and serotonin (Yoshimoto, McBride, Lumeng, & Li, 1992) signaling, and polymorphisms of the oxytocin receptor gene show differences in effects of religious priming (Sasaki, Mojaverian, & Kim, 2015), suggesting that further work is needed to identify the extent to which multifactorial neurotransmitter systems contribute to religious experience.

Theoretical models of religious cognition have proposed that networks comprised of ventral striatal and prefrontal regions may play a critical role in the development and maintenance of religious ideation. In a study of patients with Parkinson disease, who show decreased religiosity across multiple domains (Butler, McNamara, Ghofrani, & Durso, 2011), patients showed selective impairment of decreased response times following priming with religious concepts compared to neutral concepts (Butler, McNamara, & Durso, 2010). Moreover, patients with asymmetric or early impairment on the left (right hemisphere dysfunction) showed more severe impairments, suggesting that right striatal-prefrontal circuits may be most related to semantic understanding of religious concepts (Butler et al., 2010, 2011). Our results similarly showed asymmetric activation of right hemispheric attentional regions when participants reported “feeling the Spirit,” for example in Figure 3 in the anterior cingulate and frontal eye field regions.

During subjective religious phenomena, coactivation of frontal attentional regions with nucleus accumbens may be a mechanism that amplifies the subjective intensity of euphoric feelings by focused attention and alertness. The frontal eye fields, supplementary motor area, and anterior cingulate cortex are well-known regions sub-serving control of attention (Fox, Corbetta, Snyder, Vincent, & Raichle, 2006). In particular, frontal attentional regions such as the frontal eye fields and supplementary motor area participate in top-down control of attention (Corbetta & Shulman, 2002). The dorsal anterior cingulate cortex in particular may contribute to the perception of salience of religious experience (Seeley et al., 2007).

Activation of the medial prefrontal cortex in all three tasks may suggest a role in representation of affective meaning for the religious stimuli (Roy, Shohamy, & Wager, 2012) and suggests that cognitive attribution and judgment of the meaning or value of religious stimuli contributes to their experience. A role for the pregenual ventromedial prefrontal cortex in generative meaning and valuation is supported by a broad literature including studies of economic value (Chib, Rangel, Shimojo, & O’Doherty, 2009), response to highly valued immediate rewards (McClure, Laibson, Loewenstein, & Cohen, 2004), and empathic choices that involve valuation of social outcomes (Janowski, Camerer, & Rangel, 2013). A role for the ventromedial prefrontal cortex in shaping belief is also supported by evidence that neuromodulation of this regions can specifically decrease endorsement of religious beliefs (Holbrook, Izuma, Deblieck, Fessler, & Iacoboni, 2015). A growing literature suggests that medial prefrontal cortex may support social working memory load in cognitive processing (Meyer, Taylor, & Lieberman, 2015), which might be consistent with medial prefrontal activation supporting socially relevant information when participants indicated higher levels of religious experience.

Religious experience is infused with complex sociocultural features that are heterogeneous both within individuals, within any given religious community, and across faith traditions. Consequently, neural mechanisms of religious experience are likely to be multifactorial, even within individuals. While associations between reward processing regions and cortical representations may represent one important mechanism associated with religious experience, studies of other religious practices, cultures, and experiences are needed to add to a library of neural circuits associated with religious practice and spiritual experience. For example, mystical and dissociative states (Carhart-Harris et al., 2012; Peres et al., 2012) and experience associated with contemplative practices and meditation (Lutz et al., 2004; Tang et al., 2015) may involve different mechanisms than those observed in this study of a sample with shared young age, Western Christian culture, and similar religious training. Broadly, our findings are consistent with the view that religious experience may be described through known neural circuits mediating cognitive processes such as reward, social cognition, attention, and emotive processing rather than through a novel category of experience (Kapogiannis et al., 2009; Schjoedt et al., 2009).

It is possible that some of the religious and spiritual experience reported was in response to social desirability, wherein participants report greater experiences out of a desire to appear more socially consistent with the aims of the study (Edwards, 1957). This was mitigated by a study design in which each subject served as their own control, comparing for each experimental paradigm periods when feelings were less salient to time points when feelings were more salient. Nevertheless, it is possible that such influences of social desirability are reflected within our results of activated brain regions.

The relationship between ventral striatal activation and reward showed close temporal association in our data, with striatal activation preceding subjective experience of “feeling the Spirit” by 1–3 s. While it is plausible that such activation may be recognized as reward and interpreted as a component of religious experience, it remains unclear the extent to which spiritual feelings are interpreted as an interaction of multiple brain regions that contribute to the response, and activation of the nucleus accumbens after a subject decided they had experienced a spiritual response rather than associated with experience or perception of the response is not excluded. Further research may help clarify individual components of spiritual and religious experiences across individuals and across faith traditions and their relative interactions with behavior, personality, and moral cognition.

Given commonalities in brain architecture across individuals with limited neural mechanisms for representing euphoria, a search for shared neural mechanisms for intense religious and spiritual feelings across cultures may provide insight into the evolution of complex religious systems and opportunities for cross-cultural understanding of deeply held religious beliefs and experience. Ultimately, the pairing of classical reward responses with abstract religious ideation may indicate a brain mechanism for attachment to doctrinal concepts and charismatic in-group religious leaders.

## Figures and Tables

**Table 1:** Demographics, religiosity, moral values, and personality measures.

	Imaged		Not imaged		
Sex					
Female	7		8		
Male	12		17		
	**Mean**	**Std.**	**Mean**	**Std.**	***p*-Value**
Age	27.42	3.63	30.7	8.90	0.22
Years of education	15.7	2.28	15.82	2.75	0.86
Dimensions of religiosity total	20.54	1.63	20.99	1.09	0.28
Moral foundations questionnaire					
Moral foundations harm/care	21.37	4.03	19.88	4.69	0.28
Moral foundations fairness/reciprocity	18.47	4.09	17.76	4.19	0.57
Moral foundations in-group loyalty	13.58	4.65	14.12	3.68	0.67
Moral foundations authority/respect	15.95	4.22	16.00	3.45	0.96
Moral foundations purity/sanctity	22.84	4.55	21.72	4.34	0.41
NEO Five Factor Inventory (raw)					
Neuroticism	15.16	8.47	19.80	8.14	0.07
Extroversion	32.10	5.94	30.76	6.51	0.49
Openness to experience	30.42	5.40	28.88	4.82	0.32
Agreeableness	35.68	3.77	32.00	5.00	0.01
Conscientiousness	36.21	5.21	34.24	4.59	0.19

**Table 2:** Significant clusters of activation.

Hemisphere	Region	No. of voxels	*T*	*Z*	*x*	*y*	*z*	*p*_FDR-corr_
**Quotations initial**								
L	Cerebellum	3483	9.33	5.39	−12	−64	−13	<.001
R	Perirolandic	323	8.73	5.22	45	−13	38	<.001
L	Cerebellum	36	7.99	5.00	−27	−58	−55	.041
L	Entorhinal cortex	271	7.80	4.94	−15	−16	−19	<.001
R	Anterior temporal	385	6.83	4.61	42	−1	−25	<.001
L	Perirolandic	254	6.10	4.32	−42	−16	44	<.001
L	Ventral striatum	480	6.06	4.31	−6	2	−4	<.001
L	Cerebellum	39	5.89	4.24	−9	−64	−46	.038
L	Frontal pole	175	5.16	3.90	−6	53	5	<.001
**Scriptures**								
R	Superior temporal sulcus	99	7.79	4.67	48	−34	−4	<.001
L	Cerebellum	67	7.45	4.57	−27	−61	−49	.001
R	Perirolandic	255	7.35	4.54	60	2	38	<.001
L	Anterior middle frontal gyrus	260	7.19	4.49	−27	47	20	<.001
R	Anterior cingulate cortex	28	6.99	4.43	18	23	23	.048
L	Posterior superior frontal gyrus	602	6.90	4.40	−9	11	56	<.001
L	Putamen	141	6.70	4.33	−24	−22	23	<.001
L	Middle frontal gyrus	179	6.48	4.26	−42	8	50	<.001
R	Cerebellum	403	6.41	4.23	6	−52	−19	<.001
R	Cerebellum	165	6.35	4.21	30	−58	−28	<.001
L	Ventral striatum	241	6.19	4.15	−9	−7	11	<.001
R	Cerebellum	93	5.79	4.00	9	−67	−43	<.001
**Quotations final**								
	Central pons	395	7.47	4.83	0	−28	−34	<.001
L	Midbrain	56	6.57	4.51	−12	−19	−22	.012
L	Posterior inferior frontal gyrus	86	6.31	4.41	−57	8	5	.003
R	Ventral striatum	276	6.11	4.33	6	−1	−1	<.001
L	Posterior superior frontal gyrus	1031	6.00	4.28	−12	8	71	<.001
R	Superior temporal gyrus	569	5.62	4.12	51	−28	8	<.001
R	Cerebellum	66	5.51	4.07	15	−67	−34	.007
R	Posterior cingulate gyrus	218	5.45	4.04	15	−16	44	<.001
R	Cerebellum	91	5.33	3.99	39	−52	−40	.002
R	Cerebellum	61	5.05	3.85	−18	−61	−52	.009
R	Perirolandic	134	4.73	3.69	48	−1	53	<.001
R	Premotor cortex	77	4.49	3.56	24	−25	62	.004
L	Cerebellum	43	4.48	3.55	−12	−82	−16	.029
